# A multimodal screening system for elderly neurological diseases based on deep learning

**DOI:** 10.1038/s41598-023-48071-y

**Published:** 2023-11-29

**Authors:** Sangyoung Park, Changho No, Sora Kim, Kyoungmin Han, Jin-Man Jung, Kyum-Yil Kwon, Minsik Lee

**Affiliations:** 1 Department of Electrical and Electronic Engineering, Hanyang University ERICA, Ansan, 15588 South Korea; 2 Department of Neurology, Korea University Ansan Hospital, Ansan, 15355 South Korea; 3 Department of Neurology, Soonchunhyang University Seoul Hospital, Seoul, 04401 South Korea

**Keywords:** Computer science, Parkinson's disease, Stroke

## Abstract

In this paper, we propose a deep-learning-based algorithm for screening neurological diseases. We proposed various examination protocols for screening neurological diseases and collected data by video-recording persons performing these protocols. We converted video data into human landmarks that capture action information with a much smaller data dimension. We also used voice data which are also effective indicators of neurological disorders. We designed a subnetwork for each protocol to extract features from landmarks or voice and a feature aggregator that combines all the information extracted from the protocols to make a final decision. Multitask learning was applied to screen two neurological diseases. To capture meaningful information about these human landmarks and voices, we applied various pre-trained models to extract preliminary features. The spatiotemporal characteristics of landmarks are extracted using a pre-trained graph neural network, and voice features are extracted using a pre-trained time-delay neural network. These extracted high-level features are then passed onto the subnetworks and an additional feature aggregator that are simultaneously trained. We also used various data augmentation techniques to overcome the shortage of data. Using a frame-length staticizer that considers the characteristics of the data, we can capture momentary tremors without wasting information. Finally, we examine the effectiveness of different protocols and different modalities (different body parts and voice) through extensive experiments. The proposed method achieves AUC scores of 0.802 for stroke and 0.780 for Parkinson’s disease, which is effective for a screening system.

## Introduction

**Figure 1:**
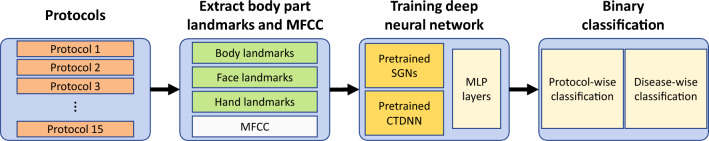
The entire process of the proposed method.

Neurological disease is one of the most common disorders, affecting the nerves found throughout the brain, body, and spinal cord. It can cause muscle weakness, seizures, paralysis, loss of sensation, and confusion. According to the Global Burden of Disease (GBD) 2015 Neurological Disorders collaborator group^1^, these are the second leading cause of death worldwide. Moreover, the number of patients requiring treatment by qualified clinicians is expected to continuously increase in the coming decades.

Among many neurological diseases, we focus on Parkinson’s disease and stroke in this study. Since the incidence of neurological diseases increases with age^2,3^, the number of patients with Parkinson’s disease and stroke is also expected to increase significantly in Korea as rapid aging is expected^4^. Parkinson’s disease, which is gradually increasing in prevalence, and stroke, which has high mortality and morbidity, need to be properly treated through early diagnosis to reduce personal, social, and national burdens. Therefore, it is important to prescreen subjects before conducting close examinations. These screening procedures must be easy, inexpensive, and efficient enough compare to existing methods.

Magnetic resonance imaging (MRI) and positron emission tomography (PET) are usually performed to identify those neurological disorders, however, these can be too costly and burdensome for screening purposes. Luckily, in the case of neurological diseases, many symptoms can be easily observed without any specialized equipment. Although a neurologist’s detailed examination is essential for an accurate diagnosis, patients may exhibit abnormal body motion or unnatural facial expressions. They can also exhibit voice disorders, such as slurred pronunciation. These symptoms can be captured relatively easily using common devices, such as video cameras. Therefore, video-based methods are effective for prescreening.

Landmarks of the human body contain information regarding physical movements and can be used to recognize various actions. Therefore, it is widely used in many intelligent applications, such as entertainment with Kinect sensors^5^, human-robot interaction^6^, and emotion recognition^7^ with facial landmarks. This demonstrates the effectiveness of landmark-based processing in acquiring high-level information on body motion. Therefore, landmark inputs can also be effective for screening neurological diseases. These landmarks, which can be detected using many existing computer vision algorithms, do not require expensive devices, such as brain PET or MRI; therefore, they can be very efficient for screening purposes. Another advantage is that the data dimension is much lower than that of RGB data, which leads to a reduction in the computational cost.

Another equally effective piece of data is voice. It is also well known that voices are good indicators of neurological diseases^8–10^ and can be recorded using the same video cameras. Voice data are one-dimensional data where the data dimension is even smaller than a sequence of landmarks; therefore, it is also efficient in terms of computational cost.

These modalities must be processed to extract high-level information for disease screening. Recently, deep learning^11^ has become popular in many fields because of its outstanding performance in finding high-level patterns in data processing. Accordingly, there have been many recent proposals for analyzing medical conditions using deep learning^12–16^. To apply deep learning to a certain problem, one must select a network structure that considers the characteristics of the underlying data.

A graph neural network (GNN) is an artificial neural network based on graphs. It can learn information about the interactions between different nodes. A human skeleton is similar to a graph in which the body landmarks are nodes, and the bones are edges. Accordingly, one can effectively model landmarks and their relationships with the GNN. The semantics-guided neural network (SGN)^17^ is an effective model for human action recognition based on landmark inputs and multistage GNNs. From the dynamic representation of human body landmarks (locations and velocities), the SGN retrieves spatiotemporal features based on a joint-level module and a frame-level module. In this study, we used pre-trained SGN models to extract high-level features of body motions.

A time-delay neural network (TDNN)^18^ is an artificial neural network used to process sequential data. It is designed to learn semantic information from sequential data, such as speech. The crossed-time delay neural network (CTDNN)^19^ is a simple but effective model for solving speaker identification problems. It was designed to obtain high-quality temporal information by fusing features from multiple TDNNs with different context sizes.

In this study, we propose a multimodal neurological disease screening model that fuses the motions of different body parts (body, face, and hands) and voice features. For this purpose, we established 15 behavioral examination protocols and built a dataset by recording them for various subjects with and without neurological conditions. The proposed deep-learning-based screening method was developed on this dataset, and it only requires video data of subjects performing simple movements or speaking simple sentences. These videos are transformed into landmark trajectories of the body, face, and hands or mel-frequency cepstral coefficients (MFCC)^19^ of voices, depending on the protocol. Then, landmark trajectories and MFCC features pass through SGNs and a CTDNN, respectively, to obtain high-level features. These features were combined to make a final decision.

SGNs for different body parts were pre-trained with appropriate datasets to extract the high-level features of their motions. The CTDNN^19^ for the voice was pre-trained with MFCC features converted from the VoxCeleb^20^ audio dataset. In addition, we trained our model not only with the disease label that indicates whether the subject is a patient or not but also with protocol labels that indicate whether a specific protocol shows possible symptoms. We designed a feature aggregator that fused the output features of the subnetworks to train the entire model. The entire process of the proposed method is shown in Fig. 1.

We verified which modality and which protocol of the input is critical through extensive experiments. We report the performance of screening for two representative neurological diseases through receiver operating characteristic (ROC) plots and the area under ROC curve (AUC) values. The proposed method achieves high AUC performance owing to the carefully designed protocols and multimodal features. The main contributions of this study are as follows:

- We propose a multimodal deep neural network (DNN) that can aggregate landmarks of different body parts and voice features for neurological disease screening.
- We propose various protocols for neurological disease screening and identify an effective setting based on extensive ablation studies.
- We report performance on two neurological diseases, Parkinson’s disease and stroke, based on multitask learning.

Our previous study^21^ investigated a similar problem but with some limitations. The limitations were threefold: (i) The study focused only on landmark data, and other modalities, such as voice, which can also be effective indicators of neurological diseases, were not considered. (ii) Only one disease (stroke) was considered. (iii) The underlying DNN structure was basic, and higher-order relations in the data could have been ignored. To overcome these limitations, in this study, we considered a multimodal system based on both landmarks and voices. In our experiments, we confirmed that voice is an effective cue and that adding it improves overall performance. We also collected a new dataset for two neurological diseases (Parkinson’s and stroke), with an increased number (15) of protocols from two hospitals. This new dataset contains more diverse conditions than the one in^21^: There was non-negligible variability in the lengths of videos because it took different times for different subjects to perform the same protocol (in^21^, this was more strictly controlled). There were also many cases where landmarks were not extracted for some body parts owing to clothing conditions such as wearing facial masks. These diverse conditions are more similar to the real environments in which the proposed method will be used. The proposed method is based on more advanced neural network structures, such as the SGNs for landmarks and CTDNN for voices, to improve the effectiveness of the underlying model. Due to the above improvements, the proposed method achieves higher performance than our previous work.

## Related works

Neurological diseases can be predicted by body movements and voices, not only by using medical equipment such as MRI or PET. The patient may tremble in certain body parts or slow down when performing an action. In addition, speaking and pronunciation may tremble or slur.

Recently, numerous machine-learning (ML)- and deep-learning (DL)-based methods have been developed to diagnose neurological diseases, thanks to their ability to learn useful features from very diverse, high-dimensional data. Many of these methods use sensors, medical images, voice, and video data. Here, we introduce related work on DL and ML methods for determining the presence of neurological diseases.

### Diagnosis with sensor data

Here, we introduce papers that collected data by attaching various specialized sensors. Eskofier et al.^22^ utilized inertial measurement unit (IMU) data by attaching two sensors to the forearms of Parkinsonians, and utilized various standard ML methods to determine the presence or absence of bradykinesia using sensor data. Pereira et al.^12^ classified Parkinson’s disease (PD) and healthy controls (HC) with CNN by obtaining handwriting dynamics using a sensor-attached pen. Maachi et al.^23^ used a public dataset^24^ collected from eight sensors placed underneath each foot to classify PD and HC through gait analysis using parallel 1D-Convnets. Um et al.^25^ classified the motor state of PD, i.e., bradykinesia and dyskinesia, using a Microsoft Band2 sensor and CNNs. Pedro et al.^26^ proposed a method for detecting the symptoms of Alzheimer’s disease and psychomotor agitation using electrocardiograms and electrodermal activity sensors and various basic ML classification methods.

The disadvantage of the above methods is that they require special sensors; therefore, they are not suitable for use in more general environments. In addition, the aforementioned studies focused only on a specific part of the body, ignoring other parts that may also contain important symptoms of neurological diseases.

### Diagnosis with image data

Here, we introduce studies incorporating ML/DL to determine neurological diseases based on medical images, such as MRI. Payan and Montana^13^ used a pre-trained sparse autoencoder and CNN on the ADNI dataset, which contains brain MRI images of HC, Alzheimer’s disease (AD), and mild cognitive impairment (MCI), for classification. Kollias et al.^27^ proposed a CNN-RNN model on 3-4 consecutive frames of brain MRI images and dopamine transporter scan images for diagnosing and predicting PD. Böhle et al.^28^ proposed layer-wise response propagation to visualize the decision process in a CNN-based classification of MRI images, so that it can provide useful information in clinical routines.

These methods can be used to help professional medical personnel diagnose diseases. However, medical equipments are requried to use these methods, which is a burden for screening purposes, as described in “Introduction”.

### Diagnosis with voice data

In this section, we introduce studies based on voice data. Wodzinski et al.^29^ used spectrograms extracted from voice data and a modified ResNet architecture to classify PD and HC based on the PC-GITA dataset^30^. Gunduz^31^ used various sets of vocal features such as recurrence time density entropy, detrended fluctuation analysis, and MFCC to find the best combination to discriminate PD and HC based on CNN. Caliskan et al.^32^ used a stacked autoencoder to classify PD and HC using two datasets containing various voice attributes, i.e., the Oxford Parkinson’s Disease Detection dataset^33^ and the multiple types of sound recordings dataset^34^.

The above studies focused on voice data and showed that they could be effective in classifying neurological diseases. However, there are other effective modalities for classifying neurological diseases, and combining them can improve performance. In this paper, we combined voice data with landmarks retrieved from visual data to improve overall performance.

### Diagnosis with landmark

Here, we introduce studies that diagnose neurological diseases or determine their symptoms based on landmark features. Bandini et al.^35^ extracted facial landmarks from videos of neutral and various emotional expressions of PD and HC subjects, and investigated the differences between PD and HC using 20 geometric features. Rajnoha et al.^36^ classified PD and HC groups using a CNN-based face embedding network based on static facial images transformed into front views using 68 facial landmarks. Li et al.^37^ used 13 body-landmark trajectories from videos of various activities to analyze motor complications and levodopa-induced dyskinesia caused by the long-term use of levodopa as a remedy for PD. Jin et al.^14^ used 106 facial landmarks in 176 records of 5-s videos collected from 64 elderly people (33 with PD). These data were used for detecting the “mask face” and judging the facial tremor based on various ML and DL methods.

These methods show that landmark features can be effective in analyzing neurological diseases. Many of the above studies have focused mainly on analyzing the symptoms of neurological diseases based on ML or DL. The others are about estimating the presence of diseases, but they mostly focus on a specific part of the body or body motion, such as face and gait. In this study, we focus on developing a multimodal screening system that combines most of the features that are easily collectible from video cameras.

**Figure 2:**
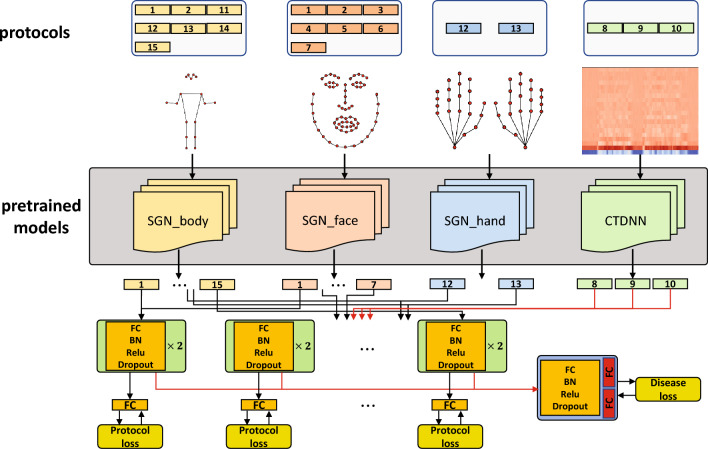
The overall structure of the proposed method. We perform binary classification for each protocol as well as for the entire data of a subject, based on the protocol label and the disease label, respectively. The weights of pre-trained SGN blocks and CTDNN in gray boxes are fixed, i.e., they are not trained in the main training procedure. For protocols where more than one body parts are used, the features are concatenated after passing through the corresponding SGNs.

## Proposed method

### Overview

In this paper, 15 protocols were designed to screen for two neurological diseases, i.e., stroke and PD. In each protocol, a subject performs a specific action or speaks a specific sentence in front of four 4K resolution cameras installed at four different positions. The details are presented in The KSSP Dataset.

Given 15 examination videos, we extracted the body, face, hand landmarks, and mel-frequency cepstral coefficients (MFCC) depending on the content of the protocol. Details of the protocols and their extracted modalities are listed in Table 1. We provide video demonstrations of these protocols performed by Dr. K., K.-Y., one of the authors of this paper, as supplementary material (Dr. K., K.-Y. provided a written informed consent regarding the publication of these video). Subsequently, we extracted high-level features from the input landmarks and MFCC using the pre-trained SGN^17^ and pre-trained CTDNN^19^, respectively. For landmarks, we extract 128-dimensional features per camera for one body part in one protocol. For voice, we extract 512-dimensional features in one protocol. After concatenating the features of the cameras (and those of different body parts if the given protocol uses more than one body part), it passes through a subnetwork to extract the features of the corresponding protocol. These features are used to estimate whether the subject performs the corresponding protocol normally with an additional FC layer.

Finally, all the subnetwork outputs are concatenated, and a feature aggregator is applied to classify the presence of diseases. This means that the subnetworks learn protocol-specific features, and the feature aggregator fuses this information to make a final decision. At the end of the feature aggregator, we perform multitask learning for stroke and PD using two separate FC layers. In this process, an HC sample passes through both the FC layers, while the stroke and Parkinsonian samples pass through only one corresponding FC. The overall process of the proposed method is shown in Fig. 2.

### The KSSP dataset

**Table 1:** Protocol description and extracted modalities.

Protocol	Description	Body	Face	Hand	Voice
1,2	Standing still and moving eyes up and down, left and right	$\checkmark$	$\checkmark$		
3	Looking up while standing still, wrinkling forehead		$\checkmark$		
4	Closing eyes tightly		$\checkmark$		
5	Showing teeth while raising the corners of mouth		$\checkmark$		
6	Opening mouth		$\checkmark$		
7	Sticking tongue out		$\checkmark$		
8	Making ‘ah’ sound				$\checkmark$
9	Speaking a sentence 1				$\checkmark$
10	Speaking a sentence 2				$\checkmark$
11	Putting both hands at chest level while palms facing the floor	$\checkmark$			
12	Pointing nose alternately with both index fingers	$\checkmark$		$\checkmark$	
13	Contacting tips of the thumb and index finger of both hands	$\checkmark$		$\checkmark$	
14	Walking through a straight line and returning back	$\checkmark$			
15	Walking through a straight line while outstretching both arms and returning back	$\checkmark$			

The clinical dataset for this study was acquired from two hospitals; Korea University Ansan Hospital (KUAH) and Soonchunhyang University Seoul Hospital (SUSH). Stroke and Parkinsonian subjects were recruited from KUAH and SUSH, respectively. The two hospitals’ institutional review boards (IRB) approved our study protocol (KUAH:2020AS0347, SUSH:2020-11-013). All the data were collected in accordance with the guidelines of the two IRBs.

The diagnosis of stroke and PD was confirmed by two neurologists, J., J.-M. in KUAH and K., K.-Y. in SUSH. The data were collected in October 2020 to December 2021, according to 15 predefined examination protocols (listed in Table 1) by collaboration between the two hospitals and Hanyang University. KUAH collected 267 subjects (62 stroke patients and 205 HC), and SUSH collected 307 subjects (63 Parkinsonians and 244 HC). Detailed descriptions of the protocols and videos are provided in supplementary materials.

In KUAH, males accounted for approximately 27%, females 73%, and 50s and 60s accounted for approximately 42% each. In SUSH, males accounted for about 32%, females 68%, and about 45%, 30%, and 20% were the 50s, 60s, and 70s, respectively. Data were collected from patients who visited or were hospitalized in the neurology departments of both hospitals. HC was collected through a clinical trial advertisement at both hospitals. Informed consent was obtained from all subjects involved in the study.

We took 4K (4096 × 2160) resolution RGB videos at 30 fps with four SONY FDR-X3000 cameras for the protocols. All protocols were performed in the standing position. Each participant’s data consisted of 15 × 4 = 60 videos taken from four different positions (center, top, left, and right) to capture information from diverse views and to minimize information loss due to self-occlusion. The center camera was installed at a height of 1000 mm from the floor and the middle of the left and right cameras. The left and right cameras were located 600 mm from the center camera on both sides. The top camera was installed 800 mm above the center camera. The landmarks of various body parts and voices were extracted from these videos.

When the instructor told the subject to perform a specific protocol and pressed a button, all cameras started recording simultaneously. When the protocol was completed, the instructor pressed the button again to stop the recording. Therefore, the videos captured by the four cameras have the same length for the same protocol of a subject. Each subject had 16 labels: one was the disease label, and the others were the protocol labels. The disease label indicates whether the subject had a neurological disease, and the 15 protocol labels indicate whether each protocol was performed normally. These clinical impressions were directly labeled by two neurologists (J., J.-M., and K., K.-Y).

The ratio between normal and abnormal cases was different for each protocol. Even if someone is a patient, there are cases in which a specific test indicates normal; similarly, there are cases where HC performs abnormally for a specific test. There were 574 subjects in our dataset, i.e., 125 patients and 449 HC. The numbers of normal and abnormal cases and the average (standard deviation) and maximum lengths of videos for each protocol are listed in Table 2. Uncertain cases are those where the protocol was not performed properly, or it was impossible to extract the face landmark or discriminate voice due to the subjects wearing facial masks.

**Table 2:** The numbers of normal and abnormal cases, average (standard deviation) and maximum frame lengths for each protocol.

Protocol	Normal	Abnormal	Uncertain	Avg. (stdv)	Max.
1	483	19	3	12.6s (4.2s)	35.0s
2	483	19	3	12.1s (4.1s)	67.5s
3	503	2	–	5.5s (2.1s)	25.0s
4	475	29	1	5.8s (2.0s)	18.0s
5	450	25	30	4.6s (2.0s)	35.5s
6	476	1	28	4.2s (1.7s)	28.5s
7	476	1	28	4.7s (1.8s)	19.5s
8	418	85	2	13.8s (2.3s)	31s
9	448	54	3	7.3s (2.4s)	38s
10	447	53	5	7.5s (1.8s)	20s
11	482	21	2	13.4s (2.3s)	47.5s
12	480	20	5	13.0s (4.3s)	39.5s
13	399	87	2	23.3s (7.5s)	47.0s
14	411	90	4	33.3s (9.4s)	141.0s
15	485	39	8	29.1s (10.0s)	124.5s

### Landmark extraction

To screen neurological disease from protocols based on body movements, we extracted landmarks of various body parts from videos for computational efficiency because we only need the information of body motion, and there is much unnecessary information, such as background, in videos.

For example, $V\in \mathbb {R}^{F \times H \times W \times C}$ denote a video, where *F* is the frame of the video and *H*, *W* and *C* are the height, width, and the number of channels (colors), respectively. In our setting, *H* is 4096, *W* is 2160, and *C* is three. Considering that some protocols end in more than 2 min, directly processing them is computationally expensive.

**Figure 3:**
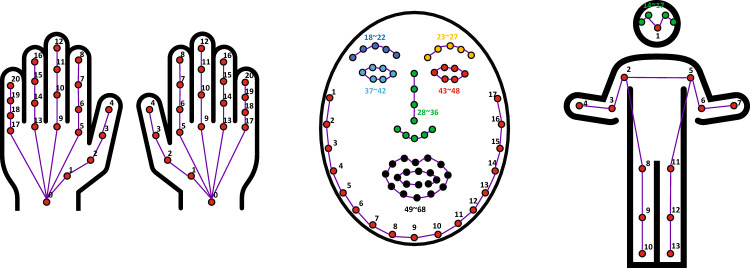
Details of landmarks. We use 13 landmarks for body, 68 for face, and 42 for hands (21 for each hand).

However, the dimension of landmark data is given by $\mathbb {R}^{F \times N \times C}$, where *N* is the number of landmarks, and *C* is the number of channels (coordinates). In our setting, *N* is no more than 68, and *C* is two. For training DNN, this can drastically reduce computational costs. The detailed numbers and locations of the landmarks for each part are shown in Fig. 3. In this study, we extracted three types of landmarks (body, face, and hand) from videos.

Furthermore, landmarks are more informative data than images in that body motions such as trembles are more distinct on landmark coordinates than on images. We downscaled the 4K videos to FHD for computational efficiency, except for the hand landmark extraction.

We used the landmark extraction framework proposed in our previous study^21^, with some modifications. First, body landmarks were extracted using AlphaPose^38^ pre-trained on the COCO keypoint detection dataset. The body contains 17 landmarks, including the nose, eyes, ears, shoulders, elbows, wrists, hips, knees, and ankles. Eye and ear landmarks were not used because they overlap with the face landmarks. Therefore, there were 13 body landmarks. Second, human faces were detected using RetinaFace^39^, and face landmarks were extracted using SAN^40^. The face landmarks include 68 landmarks of the eyes, eyebrows, nose, mouth, and edges of the face. Finally, unlike in the previous work, the sizes of the hands were relatively small in our dataset. Hence, we detected hands with Cascade R-CNN^41^ and extracted hand landmarks with MobileNetV2^42^, following the practice of^43^.

### Frame length staticizer

Many popular deep neural networks for landmark trajectories have fixed input sizes, whereas those of input samples can differ. In this case, the most commonly used methods are subsampling and padding. Subsampling reduces the number of frames to a fixed size by regularly dropping them. By contrast, padding fills the lacking frames with a meaningless value, such as zero, to increase the length.

However, in this study, momentary or small tremors and movements were very important for screening. Therefore, we could not use subsampling, which may have resulted in the loss of this information. Padding, on the other hand, does not lose any information but also has a disadvantage. If the input samples have diverse frame lengths, applying padding may increase the undesirable variability in the data, i.e., some samples will be dominated by the padded frames while others are not. Then, we must train a neural network to provide correct answers for all these different samples. This solution might still be viable if we have many training samples with diverse frame lengths. However, if the training data are relatively small, as in our case, it can be difficult to effectively handle the variability.

**Figure 4:**
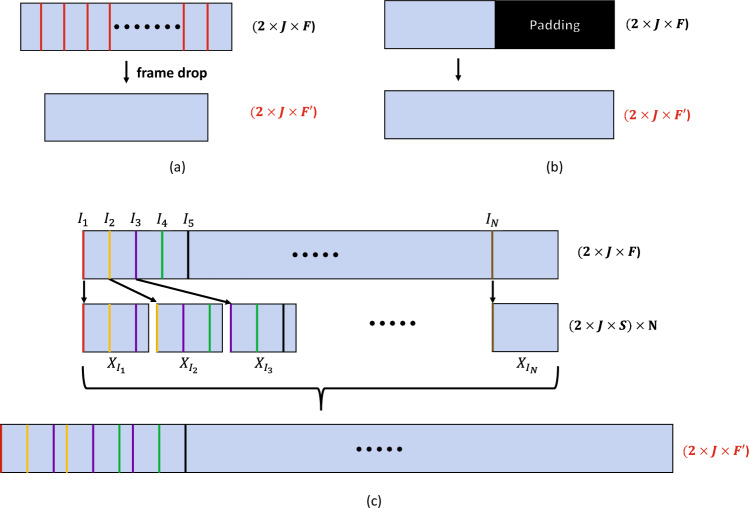
Examples of input size preprocessing. (**a**) subsampling (**b**) padding (**c**) modified frame-length staticizer. *F* and $F'$ are the number of original and staticized frames, respectively. 2 is channel dimension, *J* is number of landmarks, *I* indicates the first frames of segments *X*.

Therefore, we instead used a slightly modified version of the frame-length staticizer^44^, which we proposed in another work. This method divides a temporal sequence into several overlapping segments and outputs a fixed-length sequence by concatenating them. For inputs of different sizes, the lengths of the overlaps were adjusted to control the output size. This method does not drop frames; therefore, captures momentary information. Because each part of the output sample was a piece of the original sample, the variability of the samples was also minimized. Figure 4 compares the processes of subsampling, padding, and the modified frame-length staticizer.

The detailed procedure of the modified frame staticizer is as follows. We first determine the number of segments *N* and the number of frames *S* in one segment. These segments are concatenated on the frame axis to yield a fixed-sized output. Hence, the number of frames $F'$ in the output is $N \times S$. The detailed formula is shown below.

1 $$\begin{aligned} I_i = \lfloor (i-1)\frac{F-S}{N-1}\rfloor + 1, \quad i \in \{1,2,\cdots ,N\}, \quad X' = [X_{I_i},X_{I_{i+1}},\cdots ,X_{I_N}]. \end{aligned}$$

Here, *F* is the frame size of the original video, and $I_i$ is the starting frame of the *i*th segment. $\lfloor x \rfloor$ is the floor operation, i.e., the largest integer no more than *x*. $X_{I_i}$ indicates the *i*th segment starting from frame $I_i$. [] is frame concatenation, i.e., the segments in [] are concatenated in the frame dimension. Finally, $X'$ is the staticized video with $F'=N\times S$ frames.

With this method, the length of each sample can be fixed. Note that *S* must be smaller than the sample with the smallest frame, and $F'$ must be sufficiently large to cover all samples in our dataset. Based on these conditions, we set *N* and *S* appropriately: We used the same *N* and *S* for samples from the same body parts (even if the underlying protocol is different) to minimize the effort in pre-training, i.e., training only three SGNs. In our experiments, $F'$ for the body, face, and hands was set to 4500 (N = 150, S = 30), 1200 (N = 40, S = 30), and 1500 (N = 50, S = 30), respectively. Samples with lengths exceeding these were treated as outliers and excluded from the learning and evaluation processes. Therefore, the dimensions of the landmark sequences were as follows:

2 $$\begin{aligned} J_{body} \in \mathbb {R}^{4500 \times 13 \times 2}, \quad J_{face} \in \mathbb {R}^{1200 \times 68 \times 2}, \quad J_{hand} \in \mathbb {R}^{1500 \times 42 \times 2}. \end{aligned}$$

where $J_{body}$, $J_{face}$, and $J_{hand}$ indicate body, face, and hand landmark sequences, respectively.

### Pre-trained models

Although our data have more subjects than those of the previous work^21^ and most other neurological disorder studies, it is insufficient for training DNN. Therefore, we conducted transfer learning on the SGNs and the CTDNN. For landmark data, an SGN is pre-trained with a larger dataset for each body part to extract high-level features. Similarly, the CTDNN was trained using the VoxCeleb^20^ audio dataset for voice data. The details of the pre-training are as follows:

#### Body landmarks

 We used the NTU RGB+D60^45^ 3D skeleton dataset to pre-train an SGN for body landmarks. It contained 56,880 samples for 60 classes; some action classes had two subjects interacting with each other. Therefore, each sample consisted of 25 3D landmarks for most classes, some double that.

Because the problem in this study assumes 2D inputs, the last z-dimension was removed from this dataset, and only the landmarks of one subject were used if there were two subjects in the sample. Because our data had 13 landmarks, we reduced 25 to 13 by dropping the landmarks whose locations differed most from our data. Finally, we applied the modified staticizer to these data to obtain the same input format as that of our body landmark data. We divided the data in a cross-subject (CS) manner and trained the body SGN to classify these 60 classes. We trained the model with a batch size of 8. The classification accuracy was approximately 70%, where we applied early stopping based on test performance. This model was used as a pre-trained body model in the proposed method.

#### Face landmarks

 We used the DAiSEE^46^ dataset to pre-train the face landmarks. It is a multilabel video classification dataset that divides four emotional states (boredom, confusion, engagement, and frustration) into four levels (very low, low, high, and very high) by recording a human face. A total of 9068 video samples were obtained from 112 participants. We used 7205 and 1720 as the training and test samples, respectively. Because this dataset consists of RGB videos and not landmarks, we first detect the human face with Retinaface^39^ and extract face landmarks with^40^, similar to our face landmark data. Because each emotional label is divided by intensity, we pre-trained the face SGN through regression. We measured the performance with root mean square error (RMSE) and used the model at the epoch with the lowest RMSE value (0.47) as the pre-trained face model.

#### Hand landmarks

 We used the NVGesture dataset^47^ to pre-train the hand landmarks. It contains hand-action videos with 25 classes for driving control, 1050 training samples, and 482 test samples. Each sample contains videos of several modalities, i.e., RGB, depth, and infrared. However, because the samples in our dataset are RGB videos, we used RGB videos from this dataset to extract hand landmarks and pre-train SGN. Because the samples in this dataset contained only one hand, the dimensions of each sample were $\mathbb {R}^{F \times 21 \times 2}$. However, our dataset contains two hands; therefore, we modified the NVGesture dataset as follows. We combined the landmarks from two videos in the same class to mimic the landmarks of the two hands. The frame-length staticizer was applied to this dataset. The classification accuracy was approximately 48%, where we applied early stopping based on test performance.

#### MFCC features

**Figure 5:**
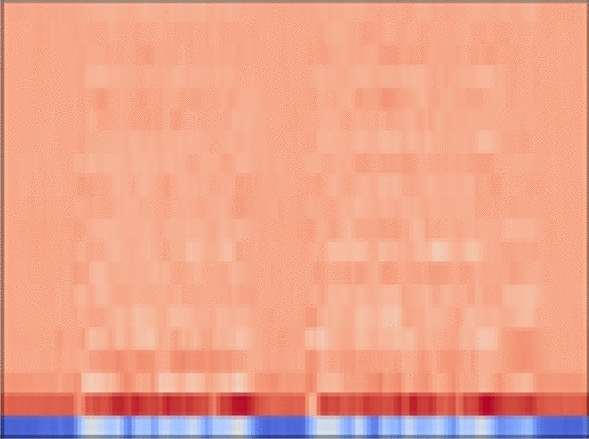
An example of MFCC features.

 We used the VoxCeleb dataset to pre-train the CTDNN. The VoxCeleb dataset consists of 74,936 audio samples from 1212 speakers, each with a different number of audio samples. To learn speaker identification based on the VoxCeleb data, we divided the audio samples into two sets for each person to construct the training and test datasets. Each audio sample was converted into MFCC features with 25 channels using the MFCC feature extraction function of the librosa library^48^. To fit the input size to our problem, the converted MFCC features were periodically cut into 2500 FFT units, considering the lengths of the samples in our dataset. An example of MFCC features is shown in Fig. 5. The final MFCC sample had dimensions of $\mathbb {R}^{2500 \times 25}$ and was utilized as an input for the CTDNN. The test accuracy of speaker identification for the VoxCeleb dataset was approximately 50.05% in our pre-training.

### Subnetworks and feature aggregator

The subnetwork is a neural network layer that transforms the features obtained from the backbone networks (SGN and CTDNN) into those that are more helpful for our tasks. Each subnetwork was composed of two blocks of (FC-BN-Relu-Dropout). The features from these subnetworks were used for disease classification and protocol-wise normality classification. The disease label and the protocol labels of a subject are not always the same because a subject may perform correctly for some protocols even if the subject has a neurological condition. Therefore, the proposed method utilizes both labels in the training procedure. For protocol-wise classification, an additional FC layer was applied to the corresponding subnetwork’s features to yield the protocol’s normality decision. For disease classification, a feature aggregator is applied to fuse the information in the features of all subnetworks.

The feature aggregator concatenates the features of the subnetworks into a single large feature vector, which is then fed into a block of (FC-BN-Relu-Dropout) and additional FC layers for multitask learning. The output of the feature aggregator is the decision for a neurological disease, which is trained based on the disease label in the KSSP dataset. The input and output channels of the layers of the subnetworks and the feature aggregator are listed in Table 3. Here, $N_s$ is given as $N_f \cdot N_c \cdot N_j$ for protocols with landmarks where $N_f$, $N_c$ and $N_j$ are the output dimension of SGNs, number of cameras, and number of body parts being used, respectively. For protocols with voice, $N_s$ is the same as the output dimension of CTDNN, i.e., 512.

**Table 3:** Details of subnetworks and feature aggregator.

	Subnetwork	Feature aggregator
Layer1	$N_s \rightarrow 64$	$8 \cdot N_p \rightarrow 8$
Layer2	$64 \rightarrow 8$	$8 \rightarrow 2$

The loss function of the proposed method is given as follows:

3 $$\begin{aligned} \mathfrak {L}_{total}=\mathfrak {L}^{s}_{f}+\mathfrak {L}^{p}_{f}+\sum _{v\in {\{1,...,15\}}}^{} \lambda _v \times \mathfrak {L}_{v}, \end{aligned}$$

The weighting of $\mathfrak {L}_{f}$, the (final) disease classification loss, was fixed at 1.0, and the weighting of $\mathfrak {L}_{v}$, the (video-wise) protocol classification loss, was set to $\lambda _v = 0.1$. The *s* and *p* in the superscripts of $\mathfrak {L}$ indicate the losses for stroke and PD, respectively. Note that the protocol labels were set to ‘uncertain’ for some samples, as shown in Table 2. In these cases, we excluded the corresponding protocol losses. In addition, because human experts labeled the protocol and disease labels in the KSSP dataset, they may contain errors that can affect the final performance of the proposed method. To mitigate this problem, we applied label smoothing^49^ to all labels for regularization. Under these conditions, we trained our network with binary cross entropy.

There was a large class imbalance in the disease labels, which was even worse for the protocol labels; i.e., many of the subjects were HC, and the majority of the examinations were normally performed. To mitigate this issue, we applied weighted sampling during training. Specifically, subjects with at least one abnormal protocol label were sampled four and six times more often than those without any for the KUAH and SUSH data, respectively. Various augmentation techniques were applied to the samples during training, the details of which are presented in “Experiments”.

## Experiments

### Experiment settings

We used threefold cross-validation for the experiments. Accordingly, the numbers of samples in the threefolds and the test set were 137, 128, 132, and 135, respectively. The data were split such that the ratios between the disease and protocol labels classes were distributed evenly for all threefolds and the test set. We reported the performance based on the AUC value. We trained the proposed model for 50 epochs and measured the validation performance for each epoch. Early stopping was applied based on the following rule: The test AUC was measured in the epoch with the highest validation AUC.

We used the Adam optimizer with a learning rate of $1\times 10^{-3}$ and weight decay of $1\times 10^{-4}$. Random rotation, scaling, and translation are applied to the landmarks in training data to minimize overfitting due to insufficient data. The rotation, scaling, and translation ranges were − 15 to 15, 70 to 150%, and − 20 to 20%, respectively. In addition, only the center and left cameras were used for landmarks to reduce overfitting, computational cost, and training time. The two cameras were used to incorporate information from diverse views and avoid possible self-occlusions. For voice, only the center camera was used because it was sufficient to capture the subject’s voice.

### Single protocol experiments

In this section, we report single protocol results to see the impacts of different protocols for each disease. In this experiment, only data from a single protocol was fed into the network, meaning that branches of the other protocols were ignored. All the other training details were identical, including that the disease label was also used in the loss function. In other words, we evaluated the significance of each protocol in the disease classification. The disease classification performance of each protocol is listed in Table 4.

**Table 4:** Test AUC of single protocol experiments.

Protocol	1	2	3	4	5	6	7	8	9	10	11	12	13	14	15
AUC$^S$	0.524	0.534	0.507	0.515	0.555	0.629	0.570	0.597	0.711	0.762	0.762	0.675	0.635	**0.774**	0.697
Protocol	1	2	3	4	5	6	7	8	9	10	11	12	13	14	15
AUC$^P$	0.569	0.573	**0.667**	0.496	0.575	0.647	0.596	0.536	0.462	0.628	0.600	0.503	0.633	0.532	0.604

*S* and *P* indicate stroke and PD, respectively. The best AUC for each neurological disease is shown in bold.

The protocol showing the best test AUC for stroke was Pro. 14, walking through a straight line and returning back. Likewise, Pro. 10 and 11, speaking a sentence and putting hands at chest level while palms facing the floor, respectively, also showed the second highest test AUC. The result indicates that these protocols exhibited more clearly distinguishable symptoms, which suggests that they can be more effective features for detecting stroke in the proposed method. Another thing to note here is that both voice (Pro. 10) and landmark data (Pro. 10 and 14) were important. Among the protocols based on body motions (Pro. 1 to 7 and Pro. 11 to 15), the ones with more dynamic motions (Pro. 11 to 15) scored higher AUC than the others (Pro. 1 to 7). The protocol showing the best test AUC performance for PD was Pro. 3, looking up while standing still, wrinkling forehead. Pro. 6, opening mouth, showed the second highest test AUC. Pro. 10, speaking a sentence, also showed a high score.

Overall, in single protocol experiments, the score of stroke was generally higher than PD. On the other hand, for protocols using face landmarks (Pro. 1 to 7), the performance of PD was better than that of stroke. Compared to Pro. 9 and Pro. 10, Pro. 8, making ‘ah’ sound, showed a relatively low test AUC. We conjecture that complex speeches provided richer information for DNN than simple vowel vocalization.

### Ablation study on various input settings

**Figure 6:**
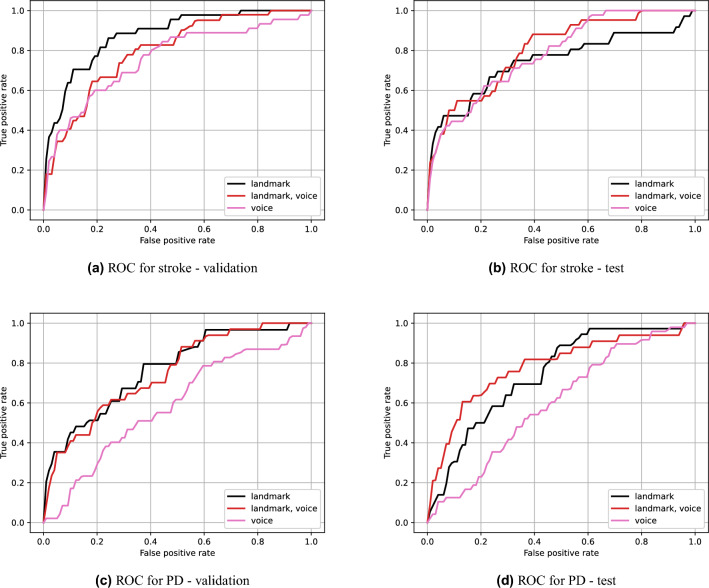
Validation and test ROC curves for different combinations of landmark and voice data. The first and second rows are for stroke and PD, respectively. The left and right columns are for validation and test performance, respectively.

In this section, we present experiments on various input settings. We primarily focus on combinations of landmark and voice data. The ROC curves are shown in Fig. 6. Table 5 summarizes the AUC values for the different configurations.

**Table 5:** Validation and test AUC results for different combinations of landmark and voice data.

Landmark	Voice	Validation AUC$^S$	Test AUC$^S$	Validation AUC$^P$	Test AUC$^P$
$\checkmark$		**0.877**	0.745	**0.768**	0.744
	$\checkmark$	0.759	0.784	0.588	0.600
$\checkmark$	$\checkmark$	0.797	**0.802**	0.751	**0.780**

*S* and *P* indicate stroke and PD, respectively. The best performance for each neurological disease is shown in bold.

Among configurations with a single modality, one with the voice features shows better performance for stroke. In contrast, for PD, that with the landmark features shows better test performance, and that with the voice features is considerably low. This result has a similar tendency to those in the single protocol experiments. Using all modalities showed the highest test AUC for both stroke (0.802) and PD (0.780), as expected. Interestingly, in terms of validation AUC, using only the landmark features showed the highest scores for both stroke and PD, but their test AUCs were not. This suggests that the multimodal approach is more effective for improving generalization performance.

**Figure 7:**
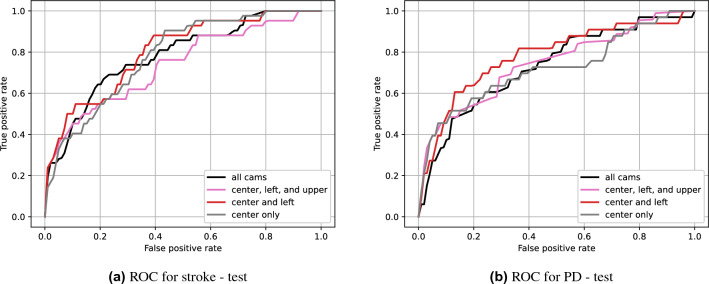
ROC curves for different combinations of cameras.

**Table 6:** Test AUC for different combinations of cameras.

	Center only	Center and left	Center, left, and upper	All cams
AUC$^S$	0.795	**0.802**	0.737	0.782
AUC$^P$	0.721	**0.780**	0.743	0.730

The best performance for each neurological disease is shown in bold.

The ROC curves for different camera combinations are shown in Fig. 7. Note that the voice data was extracted only from the center camera in all cases. Table 6 shows the corresponding test AUC. We found that using the center and left cameras was the best for both diseases. Using more than two cameras was generally worse than using one or two. We conjecture the reason to be an overfitting problem as the size of the input increases as the number of cameras increases. On the other hand, using the center and left cameras was better than the center-only scenario. This means that using more than one view can still benefit the performance, e.g., minimizing information loss due to self-occlusion, just not too many.

### Comparison to the previous work

In this section, we compare the proposed method with our previous work^21^. The previous method used only one modality (landmarks) and a much simpler backbone architecture, i.e., the landmark sequences were transformed into images using the recurrence plots (RP)^50^, which were then fed into ResNet-18^11^. A direct comparison was difficult due to different experimental settings, such as protocol design and different numbers of diseases, so we re-implemented this method for the proposed setting. Since this method is based on RP, it is referred to as the RP-based method from now on. The RP-based method used ResNet-18 as the backbone of which the output feature size was 128 for each body part. These output features were fed into the subnetworks and feature aggregator as in the proposed method. We pre-trained the backbones of the RP-based method using the same pre-training configurations for landmark data described earlier. The input RP images were constructed as follows: First, we normalized the landmark sequences using the frame length staticizer as described in (2). Then, the sequences were divided into equal-sized blocks, which were transformed into RP images and concatenated in the channel dimension. The resulting RP data for body landmarks was $250\times 250$ with 468 channels (8 blocks $\times$ 13 joints $\times$ 2 coordinates). It was $240$ with 680 channels (5 $\times$ 68 $\times$ 2) for face, and $250\times 250$ with 504 channels for hand (6 $\times$ 42 $\times$ 2). The thresholds for RP calculation were set to 0.02, 0.05, and 0.15 for body, face, and hand, respectively, which were tuned manually.

**Figure 8:**
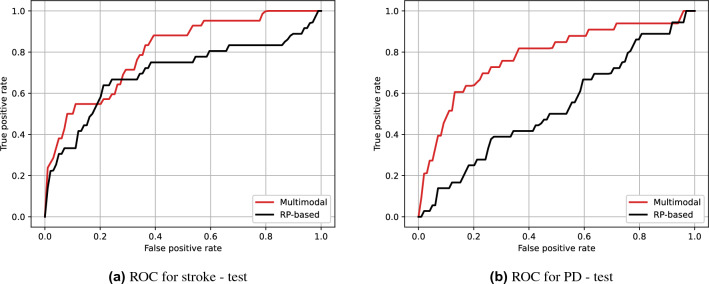
ROC curve comparison.

**Table 7:** Test AUC comparison.

AUC$^{\dagger S}$	AUC$^S$	AUC$^{\dagger P}$	AUC$^P$
0.701	**0.802**	0.531	**0.780**

$\dagger$ denotes the RP-based method.

*S* and *P* indicate stroke and PD, respectively. The best performances are shown in bold.

In Fig. 8 and Table 7, compared to the RP-based method that uses only landmark data, the proposed multimodal approach shows higher performance for both stroke and PD. The performance of the RP-based method is even worse than the landmark-only version of the proposed method in Table 5, which suggests that the proposed architecture also contributes to the performance. These results suggest that the multimodal approach, as well as the new architecture, is effective for screening neurological diseases.

## Conclusion and discussion

In this study, we proposed various examination protocols and a DNN-based framework for screening neurological diseases using various landmarks and voices. To extract high-level features from landmarks and voices, appropriate pre-training methods were applied to the backbone networks for different body parts and voices. A modified frame-length staticizer was used to preserve the important characteristics of our dataset. Through extensive experiments, we demonstrated that a multimodal approach can improve neurological disease screening. Especially, adding voice features was effective for better generalization, which confirms existing reports on the importance of voice features^8–10^. From the single-protocol experiments, we observed that dynamic motions are more effective to screen stroke than simple ones. We also found that face landmark is more effective in screening PD than stroke. The proposed method achieves the test AUCs of 0.802 and 0.780 for stroke and PD, respectively. This performance is comparable to a recent study^51^ based on the accurate measurements of hand deformities. Although the problem definition is slightly different (determining PD patients from ones already showing symptoms), another study^52^ reported a comparable performance. In other studies^53,54^, the accuracy of diagnosis ranges from 26% to 85% depending on the diagnosing conditions. Considering these related studies, we can conclude that the proposed method is reasonably effective for a screening system.

There are some studies^26,55^ achieving high performance in diagnosing specific symptoms rather than the disease itself. These methods have a different scope than ours (i.e., distinguishing possible patients from a wide variety of subjects with and without symptoms). These approaches mainly utilize accurate measurements from specialized sensors and the sample size is relatively smaller than ours (around 50 subjects). On the other hand, the proposed approach utilizes general-purpose video cameras and aggregates various anomalies to cover patients with various symptoms, as well as non-patients. Therefore, the proposed approach can be more effective for a screening system.

In future studies, conducting experiments on larger data can be important. For the proposed method, a more advanced structure based on the attention mechanism can be studied to automatically differentiate the importance of different protocols and features. In addition, addressing more diverse neurological diseases can also be important. Finally, predicting the severity of each disease is also left for future work.

### Supplementary Information

Supplementary Information.

## Data Availability

The KSSP dataset used in the current study are not publicly available due to privacy constraints. One may contact Minsik Lee (mleepaper@hanyang.ac.kr) if one has any request about the dataset.
