# Supplementary material for: A multimodal screening system for elderly neurological diseases based on deep learning
Source: Sci Rep. 2023 Nov 29;13:21013. doi: 10.1038/s41598-023-48071-y (PMC10687257; doi:10.1038/s41598-023-48071-y)
Supplement: Supplementary file 1 — Supplementary Information. [file 41598_2023_48071_MOESM1_ESM.zip › Legend_of_supplementary_videos.pdf]

Supplementary Videos

Video 1, 2. Standing still and moving eyes up and down, left and right. (repeated)

Video 3. Looking up while standing still, wrinkling forehead.

Video 4. Closing eyes tightly.

Video 5. Showing teeth while raising the corners of mouth.

Video 6. Opening mouth.

Video 7. Sticking tongue out.

Video 8. Making ‘ah' sound.

Video 9. Speaking a sentence 1.

Video 10. Speaking a sentence 2.

Video 11. Putting both hands at chest level while palms facing the floor.

Video 12. Pointing nose alternately with both index fingers.

Video 13. Contacting tips of the thumb and index finger of both hands.

Video 14. Walking through a straight line and returning back.

Video 15. Walking through a straight line while outstretching both arms and returning back.
